# Health care‐related time costs in patients with metastatic breast cancer

**DOI:** 10.1002/cam4.3461

**Published:** 2020-09-21

**Authors:** Gabrielle B. Rocque, Courtney P. Williams, Stacey A. Ingram, Andres Azuero, Stephen T. Mennemeyer, Jennifer Young Pierce, Ryan D. Nipp, Katherine E. Reeder‐Hayes, Kelly M. Kenzik

**Affiliations:** ^1^ Division of Hematology & Oncology University of Alabama at Birmingham (UAB) Birmingham AL USA; ^2^ O'Neal Comprehensive Cancer Center University of Alabama at Birmingham Birmingham AL USA; ^3^ Division of Gerontology, Geriatrics, and Palliative Care University of Alabama at Birmingham Birmingham AL USA; ^4^ School of Nursing University of Alabama at Birmingham (UAB) Birmingham AL USA; ^5^ School of Public Health University of Alabama at Birmingham (UAB) Birmingham AL USA; ^6^ Mitchell Cancer Institute University of South Alabama Mobile AL USA; ^7^ Division of Hematology & Oncology Massachusetts General Hospital Cancer Center and Harvard Medical School Boston MA USA; ^8^ Lineberger Comprehensive Cancer Center University of North Carolina Chapel Hill NC USA

**Keywords:** indirect costs, metastatic breast cancer, patient time

## Abstract

**Background:**

Burdens related to time spent receiving cancer care may be substantial for patients with incurable, life‐limiting cancers such as metastatic breast cancer (MBC). Estimates of time spent on health care are needed to inform treatment‐related decision‐making.

**Methods:**

Estimates of time spent receiving cancer‐related health care in the initial 3 months of treatment for patients with MBC were calculated using the following data sources: (a) direct observations from a time‐in‐motion quality improvement evaluation (process mapping); (b) cross‐sectional patient surveys; and (c) administrative claims. Average ambulatory, inpatient, and total health care time were calculated for specific treatments which differed by antineoplastic type and administration method, including fulvestrant (injection, hormonal), letrozole (oral, hormonal), capecitabine (oral, chemotherapy), and paclitaxel (infusion, chemotherapy).

**Results:**

Average total time spent on health care ranged from 7% to 10% of all days included within the initial 3 months of treatment, depending on treatment. The greatest time contributions were time spent traveling for care and on inpatient services. Time with providers contributed modestly to total care time. Patients receiving infusion/injection treatments, compared with those receiving oral therapy, spent more time in ambulatory care. Health care time was higher for patients receiving chemotherapeutic agents compared to those receiving hormonal agents.

**Conclusion:**

Time spent traveling and receiving inpatient care represented a substantial burden to patients with MBC, with variation in time by treatment type and administration method.

## Graphical abstract

In patients with metastatic breast cancer, time spent on health care ranged from 7% to 10% of all days included within the initial 3 months of treatment, depending on type of treatment received and its administration method. The greatest time contributions were time spent traveling for care and on inpatient services, and health care time was higher for patients receiving chemotherapeutic agents compared to those receiving hormonal agents.

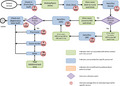

## 1 INTRODUCTION

Financial toxicity due to the direct out‐of‐pocket costs of care is central to patients' value equation,^1^, ^2^, ^3^ yet indirect costs, such as time spent receiving care, are understudied.^4^, ^5^ Such time costs can be substantial, with a previous study estimating 270 hours spent on breast cancer‐related care in the final year of life.^6^ The indirect cost of time receiving metastatic breast cancer (MBC)‐related care comes at the expense of time spent on important activities, lost work productivity, time spent with family and friends, or time spent on leisure activities.

Time‐related indirect costs are key considerations for patients when making MBC treatment decisions.^7^ Due to the increasing treatment options and varied patient preferences regarding treatment logistics,^7^, ^8^ opportunities exist for shared decision‐making for patients with MBC. Over 45 guideline‐based treatment options exist for MBC,^8^ with varying clinical time needed for administration and monitoring. Ambulatory care time may be exacerbated for patients who travel significant distances for cancer care, with our recent study finding 24% of patients with cancer traveling over an hour to receive care.^9^ Limited local cancer care resources may necessitate traveling greater distances, particularly since many community cancer clinics have closed in recent years.^10^

The primary goal of this study was to quantify the time spent on cancer care over a 3‐month period for specific medications that differ by route of administration (infusion/injection vs oral) and class (chemotherapy vs hormonal therapy). We hypothesized that (a) time spent on cancer care would be substantial; (b) patients receiving infused/injected therapy would spend more time on care than those receiving oral therapy; and (c) patients receiving chemotherapy would spend more time on care than those receiving hormonal therapy.

## 2 MATERIALS AND METHODS

### 2.1 Study design

Time spent receiving cancer care in the initial 3 months of treatment for patients with MBC was estimated using the following data sources: (a) direct observations from a time‐in‐motion quality improvement evaluation (process mapping); (b) patient surveys; and (c) administrative claims. Multiple data sources were selected due to each source's strengths in assessing a component of the time equation. Given the normal course of care would include imaging at approximately 3 months to evaluate treatment response, we restricted the analysis to the first 3 months on each drug to capture the initial treatment period without accounting for change in therapy due to progression. This study was approved by the University of Alabama at Birmingham (UAB) Institutional Review Board.

### 2.2 Data sources

#### 2.2.1 Process mapping

Patients with MBC were observed at UAB by recording time spent in cancer‐related clinical encounters from November 2016 to June 2017. Each patient was observed once. Inclusion criteria included women age ≥ 18 receiving active treatment for MBC. A convenience sample of patients was mapped to represent differing clinic visits including follow‐up, infusion, labs, bone scans, computed tomography (CT) scans, and positron emission tomography scans. For each clinical encounter, patient time was captured from clinic arrival to departure and categorized by time with each health care professional (eg, infusion nurse, front desk staff) and time at each encounter location (eg, infusion chair, waiting room).

#### 2.2.2 Patient surveys

Cross‐sectional survey data were prospectively collected on women with MBC at two Alabama academic medical centers in the Southeast to evaluate employment status, MBC‐related hours missed from work, time spent traveling from home to clinic, and time spent on cancer care‐related activities outside of clinic. Surveys were collected from June 2017 to June 2019. All women age ≥ 18 receiving treatment for MBC were eligible, which could include those who were observed for process mapping. Exclusion criteria included non‐English speakers, patients residing in nursing homes, or patients receiving hospice care. Participants received a token gift for participation.

#### 2.2.3 Administrative claims data

Administrative claims were utilized to characterize the drug‐specific frequency of specific clinical services or events (physician visits, labs, imaging). We assessed claims‐based treatments and clinical events for patients receiving MBC treatment during 2007‐2013 within the Surveillance, Epidemiology and End Results (SEER)‐Medicare linked database. Patient diagnosis data were abstracted using ICD‐9 diagnosis codes for malignant neoplasm of female breast, as well as claims for secondary metastases on at least two different dates using the provider analysis and review (MEDPAR) datafile. The patient entitlement and diagnosis summary file was also used to abstract demographic and diagnosis data. The following antineoplastic agents were selected to represent different administration methods and drug classes: fulvestrant (injection, hormonal), letrozole (oral, hormonal), capecitabine (oral, chemotherapy), and paclitaxel (infusion, chemotherapy). These medications were selected because they were the most common medication for each modality and type of therapy in the SEER‐Medicare database. Antineoplastic agents were identified using Healthcare Common Procedure Coding System, National Drug Codes, or generic drug names from national claims history, outpatient, durable medical equipment, or prescription drug event files. All treatments were received at mutually exclusive timepoints. Clinical events, including complete blood counts, complete metabolic panels, CT scans, and bone scans were captured using the same datafiles (Table S1). Inpatient hospitalizations and corresponding lengths of stay were captured using the MEDPAR datafile. Patients without complete coverage (including Part D) for the entire initial treatment period, in nursing homes, or receiving hospice care during the study period were excluded. Analyses were repeated in the Truven Health Analytics MarketScan Research Database to understand if results were similar in a younger sample.

### 2.3 Outcome: Patient health care time

**FIGURE 1:**
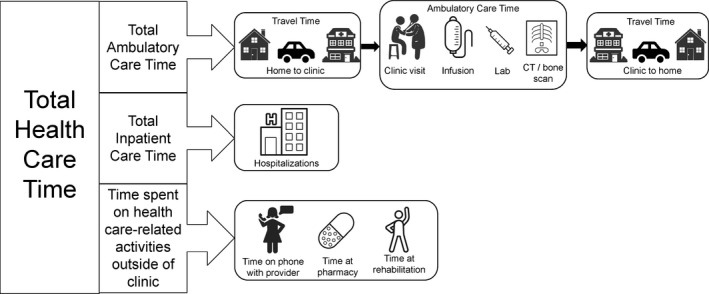
Components of time equation: Ambulatory care time, inpatient time, time spent on health care‐related activities outside of clinic

Cancer care‐related time was calculated using data from all three sources (Figure 1).^11^ Ambulatory care time, or time spent on routine care, was calculated using the following equation:

$$\begin{array}{cc} & \text{Average total ambulatory care time}\\ & \;=({\text{Average time spent traveling to and from clinic}}_{\text{survey}}\times {\text{Lab rate}}_{\text{claims}})\\ & \;+({\text{Average non-clinic visit infusion time}}_{\text{direct observation}}\times {\text{Infusion rate}}_{\text{claims}})\\ & \;+({\text{Average clinic visit time}}_{\text{direct observation}}\times {\text{Clinic visit rate}}_{\text{claims}})\\ & \;+({\text{Average lab time}}_{\text{direct observation}}\times {\text{Lab rate}}_{\text{claims}})\\ & \;+({\text{Average CT scan time}}_{\text{direct observation}}\times {\text{CT scan rate}}_{\text{claims}})\\ & \;+({\text{Average bone scan time}}_{\text{direct observation}}\times {\text{Bone scan rate}}_{\text{claims}}). \end{array}$$

This equation assumes that the patient would receive services on the same day where possible to minimize travel burden.

For patients with inpatient admissions, inpatient care time, or time spent on nonroutine care, was calculated using the following equation:

$$\begin{array}{cc} & \text{Average}\;\text{total}\;\text{inpatient}\;\text{care}\;\text{time}\\ & \;=\text{Average}\;\text{inpatient}\;\text{hospitalization}\;\text{length}\;\text{of}\;{\text{stay}}_{\text{claims}}\\ & \;\;\times \text{Inpatient}\;\text{hospitalization}\;{\text{rate}}_{\text{claims}}, \end{array}$$

Total time spent on health care was calculated using the sum of ambulatory care time, inpatient care time, and time spent on other health care‐related activities outside of clinic, which was captured from the survey data.

$$\begin{array}{cc} & \text{Average total health care}\\ & \;=\text{Average total ambulatory care time}\\ & \;+\text{Average total inpatient care time}\\ & \;+\text{Average time spent on health care−related}\;{\text{activities outside of clinic}}_{\text{survey}}, \end{array}$$

### 2.4 Statistical analysis

#### 2.4.1 All data

Descriptive statistics included means and standard deviations (SDs) or medians and interquartile ranges (IQRs) for continuous variables and frequencies for categorical variables.

#### 2.4.2 Administrative claims data

Drug‐specific event frequencies were abstracted from the claims data and used to calculate person‐month event rates during the first 3 months on treatment. Sensitivity analyses were calculated using the MarketScan Database.

## 3 RESULTS

### 3.1 Process mapping

**FIGURE 2:**
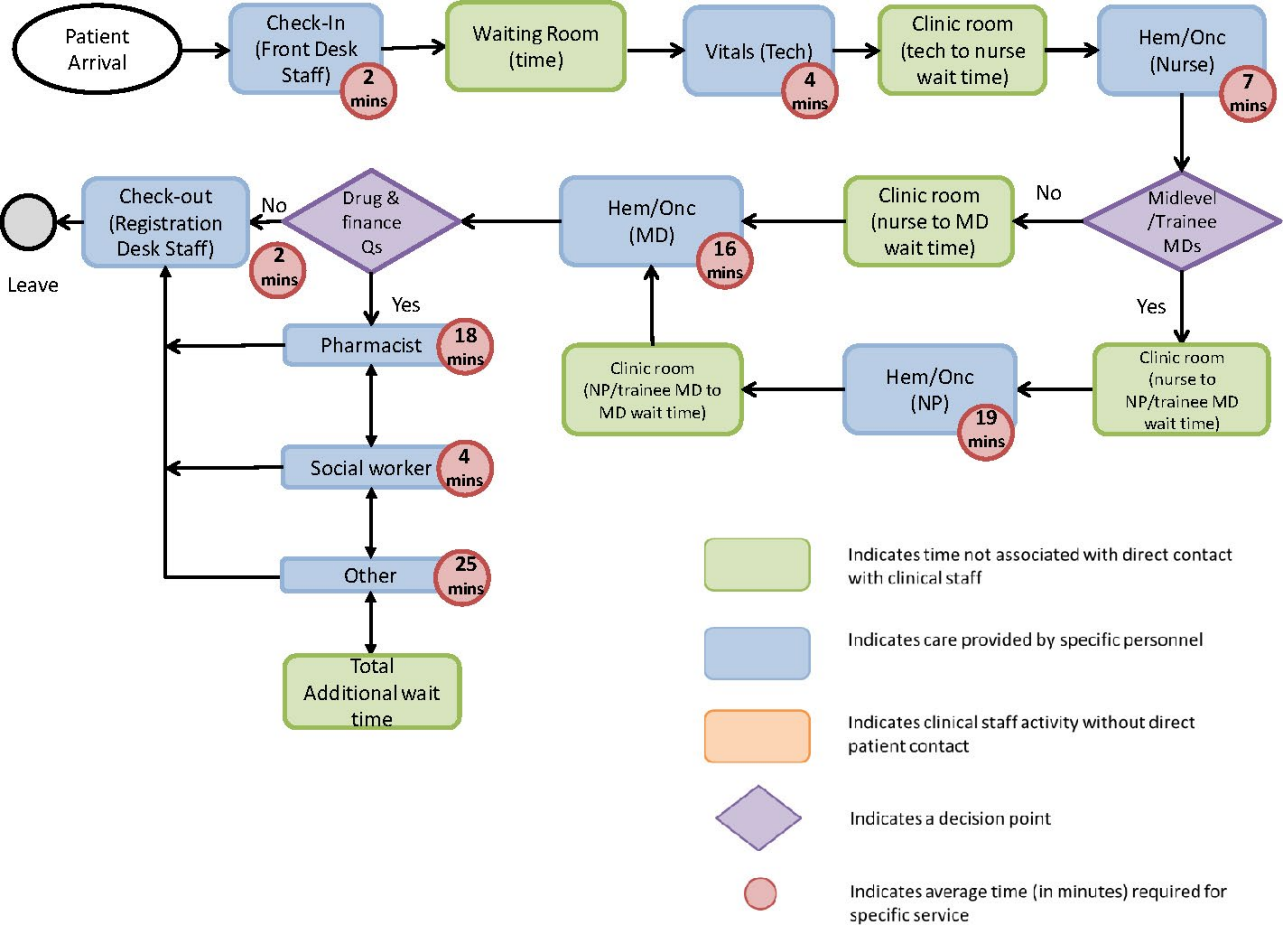
Process map: Physician follow‐up visit

We directly observed a single clinic visit for 39 patients with MBC, who received a variety of services (Table S2).These patients were a median of 58 years old (IQR 48‐65), 26% other race (not White), 85% hormone receptor‐positive, 82% human epidermal growth factor receptor 2‐negative, and 67% privately insured (Table 1).Patients were a median 12 months from metastatic diagnosis (IQR 6‐24). Figure 2 details process mapping data for a clinic follow‐up visit. On average, patients spent 4 minutes (SD 1 minutes) with their medical technician, 10 minutes (SD 10 minutes) with their nurse, 17 minutes (SD 10 minutes) with their oncologist, 18 minutes (SD 12 minutes) with their pharmacist, and 18 minutes (SD 16 minutes) with other care team members (social workers, patient navigators, chaplains). Of all shadowed patients with MBC, an average of 220 minutes (SD 105 minutes) was spent either waiting or receiving care at a typical clinical encounter, including 95 minutes (SD 44 minutes) in clinic, 123 minutes in infusion for patients receiving infusion (n = 17, SD 86 minutes), and 19 minutes (SD 23 minutes) in lab (Table 2). Process maps for visits including infusion, labs, and scans are detailed in Figures S1‐S4.

**TABLE 1:** Patient demographics and clinical characteristics for patient cohorts

	Survey	SEER‐Medicare	Shadowed
	N = 91	N = 3433	N = 39
	n (%)	n (%)	n (%)
Age (median, IQR)	58 (48‐66)	70 (65‐78)	58 (48‐65)
Race			
White	63 (69.2)	2731 (79.6)	29 (74.4)
Other	28 (30.8)	702 (20.5)	10 (25.6)
Education			
College degree	37 (40.7)	24.9%^a^	—
<College degree	50 (55.0)	75.1%^a^	—
Unknown	4 (4.4)	—	—
Income			
≥$40,000	39 (42.9)	^b^	—
<$40,000	39 (42.9)	^b^	—
Unknown	13 (14.3)	—	—
Marital status			
Single/divorced/widowed	45 (49.5)	—	12 (30.8)
Married	46 (50.5)	—	27 (69.2)
Type of metastatic disease			
*De novo*	38 (41.8)	601 (17.5)	
Secondary metastatic disease	53 (58.2)	2832 (82.5)	
Hormone receptor status			
Positive	73 (80.2)	2779 (81.0)	33 (84.6)
Negative	17 (18.7)	327 (9.5)	5 (12.8)
Unknown	1 (1.1)	327 (9.5)	1 (2.6)
HER2 status			
Positive	26 (28.6)	90 (2.6)	6 (15.4)
Negative	61 (67.0)	430 (12.5)	32 (82.1)
Unknown	4 (4.4)	2913 (84.9)	1 (2.6)
Insurance status			
Private	41 (45.1)	—	26 (66.7)
Medicare	36 (39.6)	2130 (62.0)	9 (23.1)
Medicaid	14 (15.4)	1303 (38.0)^c^	4 (10.3)

Abbreviations: HER2, human epidermal growth factor receptor 2; IQR, interquartile range.

^a^ Census track percent education.

^b^ Census track poverty level of ≥ 20% = 21.7%.

^c^ Dual eligible with Medicare n (%).

**TABLE 2:** Drug‐specific time spent to, in, and from oncology clinic visit during 3 mo on treatment for metastatic breast cancer patients in the SEER‐Medicare database

	Average minutes	Rate: Letrozole	Rate: Fulvestrant	Rate: Capecitabine	Rate: Paclitaxel
Process mapping data					
Clinic visit time	95				
Lab time	19				
Infusion only (nonclinic visit) time	123				
Bone scan time	134				
Computed tomography scan time	68				
Survey data					
Time spent traveling to/from clinic	154.5				
Weekly time spent on health care‐related activities outside of clinic	66				
SEER‐Medicare data					
Clinic visit rate		4.9	5.5	6.1	8.9
Lab rate		3.3	3.5	4.8	9.8
Infusion rate		—	3.9	—	7.9
Bone scan rate		1.0	1.0	0.7	1.1
Computed tomography scan rate		1.2	1.2	1.3	1.4
Inpatient hospitalization rate		0.8	1.3	1.3	1.2
Inpatient hospitalization length of stay		6.2 d	4.7 d	5.1 d	5.1 d
Time calculations					
Travel time = (Time spent traveling to and from clinic × Lab rate)		509.9 min	540.8 min	741.6 min	1514.1 min
Infusion time = (Nonclinic visit infusion time × Infusion rate)		0 min	479.7 min	0 min	971.7 min
Total clinic visit time = (Clinic visit time × Clinic visit rate)		465.5 min	522.5 min	579.5 min	845.5 min
Total lab time = (Lab time × Lab rate)		62.7 min	66.5 min	91.2 min	169.1 min
Total computed tomography scan time = (CT scan time × CT scan rate)		81.6 min	81.6 min	88.4 min	95.2 min
Total bone scan time = (Bone scan time × Bone scan rate)		134 min	134 min	93.8 min	147.4 min
Total ambulatory care time		20.9 h	30.4 h	26.6 h	62.4 h
Time spent on health care‐related activities outside of clinic		13.2 h	13.2 h	13.2 h	13.2 h
**Total health care time (routine)**		**34.1 h (1.4 d)**	**43.6 h (1.8 d)**	**39.8 h (1.7 d)**	**75.6 h (3.1 d)**
Total inpatient care time = (Inpatient hospitalization length of stay × Inpatient hospitalization rate)		119.0 h (7142.4 min)	146.6 h (8798.4 min)	159.1 h (9547.2 min)	146.9 h (8812.8 min)
**Total health care time if hospitalized (nonroutine)**		**153.1 h (6.4 d)**	**190.2 h (7.9 d)**	**198.9 h (8.3 d)**	**222.5 h (9.3 d)**

### 3.2 Patient survey

Of 143 patients approached for survey participation, 132 (92%) consented. Of consenting patients, 100 (76%) completed the survey; 9 were excluded due to missing data, resulting in a final sample of 91 patients. Of respondents, median age was 58 (IQR 48‐66), 31% were Black, 41% held a college degree, and 43% had a household income of <$40,000 (Table 1).Most patients were retired (29%), 15% worked full‐time, 8% worked part‐time, and 20% were on disability. Patients were a median 24 months from metastatic diagnosis (IQR 10‐39). In an average week, patients reported spending a mean of 77 minutes (SD 59), traveling from their home to clinic and a mean of 156 minutes per week (SD 106) receiving care at their clinic visit. Patients spent a mean of 66 minutes (SD 99) in an average week on cancer care‐related activities outside of clinic (eg, travel to pharmacy, calls with nurse, physical therapy). For working women, a mean of 10 hours (SD 13) was missed from work during an average week.

### 3.3 Administrative claims

patient samples, since women

Rates of clinical events during the initial 3 months of treatment are shown in Table 2. Higher per person rates of lab events in the first 3 months were found in patients receiving chemotherapy (capecitabine = 4.8, paclitaxel = 9.8) compared to those receiving hormonal therapy (fulvestrant = 3.5, letrozole = 3.3). Infusion rates were also higher for infusion chemotherapy in the initial 3 months of treatment (paclitaxel = 7.9) compared to infusion/injection hormone therapy (fulvestrant = 3.9). Similar rates of scans were found in patients receiving differing therapy types, with about one of each imaging test in the initial 3 months from treatment initiation. Rates of clinical events in the MarketScan database were similar to those in the SEER‐Medicare database (Table S3).

### 3.4 Estimation of total health care time: Combined results from all data sources

During the initial 3 months of treatment, estimated time spent on ambulatory care was highest for patients receiving weekly infused paclitaxel chemotherapy, (62.4 hours) and lowest for patients on oral letrozole hormonal therapy (20.9 hours; Table 2). Patients receiving injection/intravenous treatments, compared with those receiving oral therapy, spent more time in ambulatory care for both hormonal (fulvestrant = 30.4 hours vs letrozole = 20.9 hours) and chemotherapy (paclitaxel = 62.4 hours vs capecitabine = 26.6 hours). The greatest contribution to time spent on ambulatory care was travel time (30%‐47% of total). This was notably greater for treatments that required more frequent ambulatory care visits, such as paclitaxel. In contrast, ambulatory care time spent with a physician represented only 23%‐37% of total ambulatory care time. After incorporating time spent on health care‐related activities outside of clinic, total time receiving routine health care during the initial 3 months of treatment ranged from 34.1 hours for patients receiving letrozole to 75.6 hours for patients receiving paclitaxel.

Overall, 12% of patients had an inpatient hospitalization during their 3‐month timeframe, including 13% receiving letrozole, 9% receiving fulvestrant, 12% receiving capecitabine, and 17% receiving paclitaxel. Inpatient admissions were primarily treatment related, with shortness of breath and fever being the most frequent reasons for admissions (both 5% of total admissions). For hospitalized patients, average total time spent on health care including nonroutine inpatient care ranged from 6.4 to 9.3 days, which is 7%‐10% of all days included within the initial 3 months of treatment. Total health care time was dominated by their inpatient admission, which represented 78%, 77%, 80%, and 66% of total health care time for patients receiving letrozole, fulvestrant, capecitabine, and paclitaxel, respectively. Similar inpatient hospitalization rates and average length of stay (letrozole 6.2 days, fulvestrant 4.7 days, capecitabine 5.1 days, paclitaxel 5.1 days) were found for these four common treatments.

## 4 DISCUSSION

In this study of care time among women with MBC, we estimated an average of 1.4‐3.1 days spent traveling to, waiting for, and receiving cancer care during the initial 3 months of treatment. Relatively little time was spent communicating in‐person with a provider. Time varied by treatment regimen, with infused/injected antineoplastic therapies requiring greater time commitments than oral treatment and chemotherapies more than hormonal therapies. In this study, patients spent an average of 220 minutes at a typical clinical encounter. These estimates are greater than previously reported estimates by Yabroff and colleagues, who utilized Medical Expenditure Panel Survey data to construct ambulatory time estimates for patients with cancer.^4^ While differences may reflect clinic variability or differences in data collection methods (survey vs direct observation), the overall ambulatory time of more than 3 hours in either study may come as a surprise to oncologists who spend approximately 20 minutes with patients in a typical clinic encounter.

The greatest component of patient ambulatory care time was not time with the oncologist, but rather time spent traveling. Previous literature has reported associations between increased distance and time traveled to care for patients with more advanced disease stages.^12^, ^13^, ^14^, ^15^, ^16^, ^17^, ^18^ However, our findings demonstrated a considerably higher travel time of 155 minutes compared with that reported by Yabroff et al, who reported average travel times of 35‐39 minutes.^4^ This may reflect differences in the patient samples, since women with MBC living in the Deep South may travel greater distances for specialized treatment at a tertiary care center compared to a nationally representative sample of patients with cancer treated in both local and tertiary care environments. Our study also highlights the impact of clinic visit frequency on travel time, even for patients with shorter travel distances. This component contributed to observed differences in time spent on health care across different treatment modalities and types, with infusion therapy leading to a greater burden on patient indirect costs than oral therapies and greater time for patient on chemotherapy than hormonal therapies. This is likely related to more frequent encounters with the health care system due to higher rates of lab and clinical monitoring for patients receiving infusion/injection chemotherapy treatment. Thus, due to the potential implications on daily life, oncologists should consider both treatment type and modality when discussing treatment options with patients. Furthermore, clinicians in tertiary care settings should consider recommending local providers for monitoring and treatment where possible.

For the one in nine patients anticipated to have an inpatient admission during their initial 3 months on treatment, we found a strikingly high proportion of time attributed to their admission, which is infrequently considered in traditional cost evaluation models. Specifically, hospitalizations accounted for 66%‐80% of patients’ total time spent on health care. Importantly, hospitalization rates were similar across treatments, which may reflect a population who is often admitted for symptom management or complications of disease, rather than complications of treatment. While hospitalizations occurring during cancer care are frequently targeted in payment reform efforts, such as the Oncology Care Model,^19^ little emphasis has been placed on indirect costs or productivity losses for patients hospitalized during treatment. Given the substantial impact on patient time, efforts to reduce hospitalization should be considered.

Findings should be interpreted in the context of several limitations. First, three separate samples are presented to generate estimates, which differ by patient characteristics including age and race. For process mapping and patient surveys, we expect differences in parameter estimates to be modest for patients of different clinical and disease characteristics. For the SEER‐Medicare analysis, we acknowledge the predominately older adult sample. Estimates from Yabroff and colleagues demonstrate that similar chemotherapy visit rates (8.5 vs 9.0) and slightly higher outpatient/office visit rates (11.1 vs 13.9) are found when comparing patients aged 18‐64 and 65 years and older.^4^ Given our use of the SEER‐Medicare database to identify rate estimates for the total time calculation, we may overestimate the time spend in clinics for those <65 years of age. However, estimates based on MarketScan data, a sample which includes patients <65 years old, revealed only modest differences. These are national data sets, whereas the patient survey data include two academic medical centers in the Deep South and represent a snapshot of a single visit rather than all time spent over the initial 3‐month period. Although patients were on different treatments, we believe that time with physicians is more likely linked to clinic template time availability (eg, 20‐minute block for a follow‐up visit) rather than patient or treatment characteristics. We assumed in this analysis that patients would receive the services on the same day, which may underestimate time if services were provided on different days. In both claims and observational data, some nononcology care may have been captured. However, this overlap is representative of real‐life clinical practices and further highlights a need to coordinate care. We recognize that other patient‐specific factors (eg, attitude toward time consciousness, working status), disease‐related factors (eg, symptoms from disease), treatment‐related factors (eg, length of time for specific infusions), and center‐related factors (eg, efficiency of cancer center, availability of home‐based resources) are not accounted for in this analysis and could impact individual patient time spent on care. These estimates are intended to guide discussion but should be tailored for individual patients. We also acknowledge that SEER‐Medicare codes for secondary metastases are unlikely to be complete and accurate due to lack of association with payment. Thus, this approach to identification of patients with MBC will not fully capture this population. However, we believe that this approach still provides a reasonable estimate of frequency of treatments and health care utilization for patients with breast cancer receiving these common regimens. The data sources are from disparate times and using different methodology due to data availability. However, we anticipate only a minor impact on our estimates due to consistent usage and surveillance of these specific medications over the last decade. Of note, this study does not include novel regimens, such as hormonal blockade with cyclin‐dependent kinase 4/6 inhibitors, because these were not approved during the study period. However, based on this methodology, we would anticipate the additional monitoring associated with these regimens would result in time estimates similar to capecitabine, the oral chemotherapy agent modeled in this study. We acknowledge that this study does not account for caregiver time. Finally, our study did not attempt to determine the monetary value of patient time.

Despite limitations, this study provides the important estimates of time spent in the health care system for patients receiving treatment for MBC. Furthermore, this study emphasizes the importance of capturing time using multiple sources, including direct observation and surveys, to obtain first‐hand information on patient time in care. The process maps created in this study can be utilized as a framework for future studies examining indirect costs associated with receiving cancer care and easily adapted to meet specific hospital systems. Future work should consider the impact on patient time throughout the MBC disease trajectory, as well as the relationship between patient time spent on MBC treatment and overall survival.

## 5 CONCLUSION

Patients with MBC spend 7%‐10% of their time on health care in the initial 3 months of treatment, with differences noted by treatment type and method of administration. Travel time and time spent receiving inpatient care represented a substantial time burden to patients. Indirect costs associated with the time spent on health care are not trivial and should be considered when defining value for patients with MBC.

## CONFLICT OF INTEREST

Dr. Rocque received research funding from Pfizer, and Carevive and consulting fees and travel from Genentech and Pfizer unrelated to this manuscript. Dr. Rocque is also supported by an American Cancer Society Mentored Research Scholar Grant (MRSG‐17‐051‐01‐PCSM). No other authors have conflicts to declare.

## AUTHOR CONTRIBUTIONS

GBR, CPW, STM, KMK, and RDN were involved in conceptualization and design: GBR, CPW, SAI, AA, JYP, KRH, and KMK were involved in formal analysis and investigation. All authors were involved in writing—original draft preparation and writing—review and editing. GBR was involved in funding acquisition. GBR and SAI were involved in resources.

## Supporting information

Supplementary Material

## Data Availability

The data that support the findings of this study are available from the corresponding author upon reasonable request.
