# Supplementary material for: Health care‐related time costs in patients with metastatic breast cancer
Source: Cancer Med. 2020 Sep 21;9(22):8423–31. doi: 10.1002/cam4.3461 (PMC7666754; doi:10.1002/cam4.3461)

Supplemental Table 1. Codes used to identify clinical events.

| **Clinical category** | **Clinical event** | **Codes** |
| --- | --- | --- |
| Antineoplastic agent | Fulvestrant | HCPCS: J9395  Generic drug name: FULVESTRANT |
|  | Letrozole | Generic drug name: LETROZOLE |
|  | Capecitabine | HCPCS: J8520, J8521  Generic drug name: CAPECITABINE |
|  | Paclitaxel | HCPCS: J9264, J9265, J9267 |
| Labs | Complete blood count | HCPCS: 85004, 85007, 85008, 85009, 85013, 85014, 85018, 85025, 85027, 85032, 85041, 85044, 85045, 85046, 85048, 85049, G0306, G0307 |
|  | Metabolic panel | HCPCS: 80047, 80048, 80053 |
| Scans | Bone | HCPCS: 78300, 78305, 78306, 78315, 78320, 78399 |
|  | Computed tomography | HCPCS: 70450, 70460, 70470, 70480-70482, 70486-70488, 70490-70492, 70496, 70498, 71250, 71260, 71270, 71275, 72125-72133, 72191-72194, 73200-73202, 73206, 73700-73702, 73706, 74150, 74160, 74170, 74174-74178, 75571-75574, 75635, 76380, 76497 |
|  | Positron emission tomography | HCPCS: 78811-78816, 78608, 78609, 78459, 78491, 78492 |
| Clinic visits | Physician evaluation and management | HCPCS: 99201-99205, 99211-99215 |

HCPCS=Healthcare Common Procedure Coding System

Supplemental Table 2. Number of patients observed by type of health service (N=39).

| **Type of health service** | **Number of patients observed** |
| --- | --- |
| Clinic visits | 37 |
| Labs | 32 |
| Computed tomography scans | 11 |
| Bone scans | 11 |
| Infusions | 21 |

Supplemental Table 3. Ambulatory and non-ambulatory care rates during first three months of treatment for included drugs in the (A) SEER-Medicare and (B) MarketScan databases.

| **(A) SEER-Medicare Database** | | | | |
| --- | --- | --- | --- | --- |
| **Drug** | **Number of Events** | **Number of**  **Patient-Months** | **Rate/Person/3 Months** | **Median Number**  **of Events (IQR)** |
| Clinic Visits | | | | |
| Fulvestrant | 3298 | 1794 | 5.5 | 3 (2-5) |
| Letrozole | 3197 | 1962 | 4.9 | 3 (2-5) |
| Capecitabine | 1021 | 504 | 6.1 | 4 (2-6) |
| Paclitaxel | 5908 | 1989 | 8.9 | 5 (3-8) |
| Labs (complete blood count or metabolic panel) | | | | |
| Fulvestrant | 2049 | 1755 | 3.5 | 2 (1-4) |
| Letrozole | 2024 | 1821 | 3.3 | 2 (1-4) |
| Capecitabine | 780 | 492 | 4.8 | 3 (2-5) |
| Paclitaxel | 6474 | 1992 | 9.8 | 5 (3-9) |
| Infusion | | | | |
| Fulvestrant | 2248 | 1749 | 3.9 | 4 (3-4) |
| Letrozole | -- | -- | -- | -- |
| Capecitabine | -- | -- | -- | -- |
| Paclitaxel | 5231 | 1992 | 7.9 | 9 (5-10) |
| Scans: Computed tomography | | | | |
| Fulvestrant | 192 | 462 | 1.2 | 1 (1-1) |
| Letrozole | 230 | 564 | 1.2 | 1 (1-1) |
| Capecitabine | 92 | 207 | 1.3 | 1 (1-1.5) |
| Paclitaxel | 438 | 966 | 1.4 | 1 (1-2) |
| Scans: Bone | | | | |
| Fulvestrant | 74 | 216 | 1.0 | 1 (1-1) |
| Letrozole | 71 | 207 | 1.0 | 1 (1-1) |
| Capecitabine | 14 | 57 | 0.7 | 1 (1-2) |
| Paclitaxel | 74 | 204 | 1.1 | 1 (1-1) |
| Scans: Positron emission tomography | | | | |
| Fulvestrant | 96 | 276 | 1.0 | 1 (1-1) |
| Letrozole | 105 | 306 | 1.0 | 1 (1-1) |
| Capecitabine | 44 | 120 | 1.1 | 1 (1-1) |
| Paclitaxel | 152 | 435 | 1.0 | 1 (1-1) |
| Inpatient hospitalizations | | | | |
| Fulvestrant | 141 | 321 | 1.3 | 1 (1-1) |
| Letrozole | 109 | 393 | 0.8 | 1 (1-1) |
| Capecitabine | 100 | 231 | 1.3 | 1 (1-1) |
| Paclitaxel | 154 | 375 | 1.2 | 1 (1-1) |
| **(B) MarketScan Database** | | | | |
| **Drug** | **Number of Events** | **Number of**  **Patient-Months** | **Rate/Person/3 Months** | **Median Number**  **of Events (IQR)** |
| Clinic Visits | | | | |
| Fulvestrant | 35894 | 20907 | 5.2 | 3 (2-5) |
| Letrozole | 34525 | 22620 | 4.6 | 3 (2-5) |
| Capecitabine | 39529 | 20523 | 5.8 | 3 (2-6) |
| Paclitaxel | 68393 | 26121 | 7.9 | 5 (2-8) |
| Labs (complete blood count or metabolic panel) | | | | |
| Fulvestrant | 23704 | 17775 | 4.0 | 3 (2-4) |
| Letrozole | 21406 | 17316 | 3.7 | 3 (1-4) |
| Capecitabine | 30147 | 17439 | 5.2 | 3 (2-5) |
| Paclitaxel | 78912 | 24084 | 9.8 | 6 (3-9) |
| Infusion | | | | |
| Fulvestrant | 26954 | 20739 | 3.9 | 4 (3-5) |
| Letrozole | -- | -- | -- | -- |
| Capecitabine | -- | -- | -- | -- |
| Paclitaxel | 63829 | 24492 | 7.8 | 9 (5-10) |
| Scans: Computed tomography | | | | |
| Fulvestrant | 2726 | 6408 | 1.3 | 1 (1-1) |
| Letrozole | 3134 | 7440 | 1.3 | 1 (1-1) |
| Capecitabine | 3865 | 8811 | 1.3 | 1 (1-1) |
| Paclitaxel | 5482 | 12267 | 1.3 | 1 (1-2) |
| Scans: Bone | | | | |
| Fulvestrant | 1056 | 2988 | 1.1 | 1 (1-1) |
| Letrozole | 1119 | 3228 | 1.0 | 1 (1-1) |
| Capecitabine | 968 | 2700 | 1.1 | 1 (1-1) |
| Paclitaxel | 1141 | 3204 | 1.1 | 1 (1-1) |
| Scans: Positron emission tomography | | | | |
| Fulvestrant | 1235 | 3537 | 1.0 | 1 (1-1) |
| Letrozole | 1307 | 3753 | 1.0 | 1 (1-1) |
| Capecitabine | 1277 | 3645 | 1.1 | 1 (1-1) |
| Paclitaxel | 1834 | 5190 | 1.1 | 1 (1-1) |
| Inpatient hospitalizations | | | | |
| Fulvestrant | 514 | 1242 | 1.2 | 1 (1-1) |
| Letrozole | 527 | 1176 | 1.3 | 1 (1-2) |
| Capecitabine | 734 | 1767 | 1.2 | 1 (1-1) |
| Paclitaxel | 1149 | 2625 | 1.3 | 1 (1-1) |

IQR=interquartile range

Supplemental Figure 1. Process map: Infusion with physician visit.


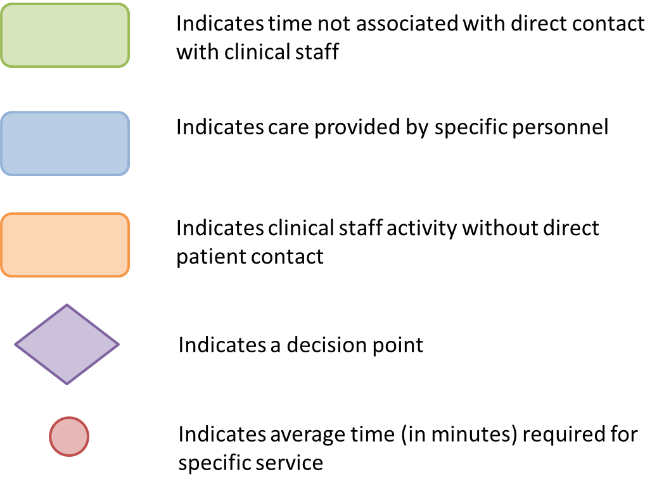


Supplemental Figure 2. Process map: Lab visit.


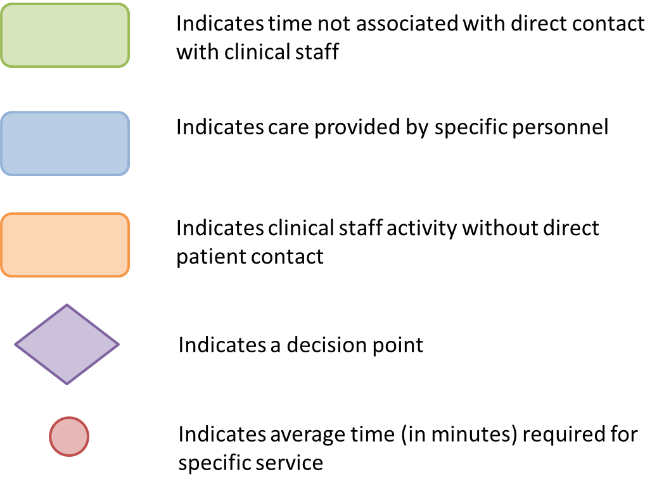


Supplemental Figure 3. Process map: Bone scan.


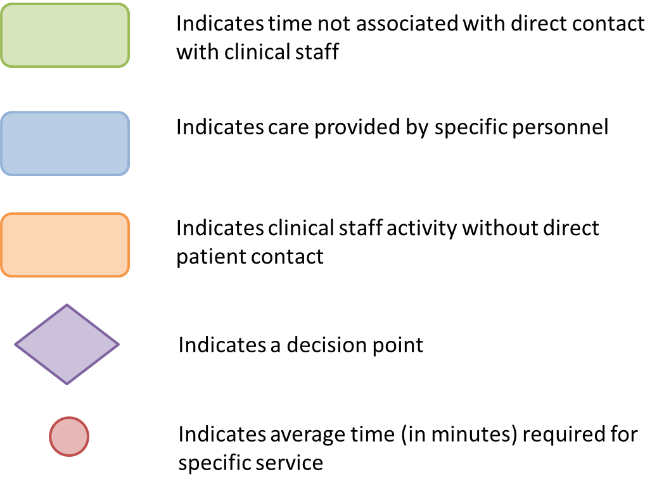


Supplemental Figure 4. Process map: Computed tomography (CT) and positron emission tomography (PET) scan.


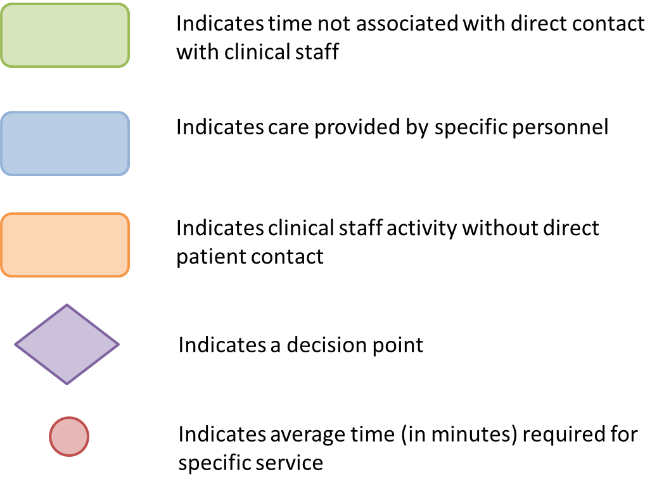

Supplement: Supplementary file 1 — Supplementary Material [file CAM4-9-8423-s001.docx]
